# Differential protein expression in endothelial cells exposed to serum from patients with acute graft‐vs‐host disease, depending on steroid response

**DOI:** 10.1111/jcmm.17712

**Published:** 2023-04-04

**Authors:** Julia Martinez‐Sanchez, Marta Palomo, Alexandra Pedraza, Ana Belén Moreno‐Castaño, Sergi Torramade‐Moix, Montserrat Rovira, María Queralt Salas, Joan Cid, Gines Escolar, Olaf Penack, Enric Carreras, Maribel Diaz‐Ricart

**Affiliations:** ^1^ Josep Carreras Leukaemia Research Institute Hospital Clínic de Barcelona, Universitat de Barcelona Barcelona Spain; ^2^ Hemostasis and Erythropathology Laboratory, Hematopathology, Department of Pathology, Centre de Diagnòstic Biomèdic (CDB) Hospital Clínic de Barcelona, Institut d'Investigacions Biomèdiques August Pi i Sunyer (IDIBAPS), Universitat de Barcelona Barcelona Spain; ^3^ Barcelona Endothelium Team Barcelona Spain; ^4^ Blood Bank Department Hematopoietic Transplantation Unit, Banc de Sang i Teixits, Hospital Clínic Barcelona Spain; ^5^ Hematology Department Bone Marrow Transplantation Unit, Institut Clínic de Malalties Hemato‐Oncològiques (ICMHO), Hospital Clínic Barcelona Spain; ^6^ Apheresis & Cellular Therapy Unit, Department of Hemotherapy and Hemostasis ICMHO, Hospital Clínic de Barcelona, IDIBAPS, Universitat de Barcelona Barcelona Spain; ^7^ Hematology, Oncology and Tumorimmunology Department Charité‐Universitätsmedizin Berlin, Freie Universität Berlin and Humboldt‐Universität zu Berlin Berlin Germany

**Keywords:** aGVHD, endothelial damage biomarkers, endothelium, proteomic analysis, steroid‐refractory aGVHD

## Abstract

Graft‐versus‐host disease (GVHD) is a complication of allogeneic haematopoietic cell transplantation. Endothelial injury is crucial as pathophysiological substrate for GVHD. GVHD first‐line treatment is high‐dose corticosteroids, although some patients are steroid‐refractory. Through the present study, we compared the endothelial proteomic profiles in response to serum from steroid‐refractory acute GVHD (SR‐aGVHD) and steroid‐sensitive acute GVHD (SS‐aGVHD) patients. Blood samples from SR‐aGVHD (*n* = 4) and SS‐aGVHD (*n* = 8) patients were collected at aGVHD diagnosis. Endothelial cell cultures were exposed (48 h) to patients' serum. Protein extraction and proteomic analysis were performed. Differences were statistically evaluated by multivariate analysis. Forty‐four proteins contributed to separate all samples into the two study groups, among which 15 participated significantly (*p* < 0.05), 10 exhibiting a fold change >1.2. Differentially expressed proteins were mainly associated with oxidative phosphorylation (Cytochrome C oxidase subunit 6B1, CX6B1), inflammation and angiogenesis (Apolipoprotein D, APOD), cell survival (Rapamycin‐insensitive companion of mTOR, RICTR), and oxidative stress (Riboflavin kinase, RIFK). This pilot study used a novel approach to distinguish the aGVHD response to steroid treatment. The proteins differentially expressed could constitute potential biomarkers for steroid‐treatment response. These findings signify a step forward to identify the mechanisms of response to steroids, of high clinical relevance considering the SR‐aGVHD elevated mortality.

## 1 INTRODUCTION

Haematopoietic stem cell transplantation (HCT) is a well‐established treatment for several haematologic, metabolic and neoplastic disorders,^1^ but it is not exempt from complications that appear early after the procedure. Acute graft‐versus‐host disease (aGVHD) is the main complication of allogeneic HCT (allo‐HCT) and remains one of the major causes of mortality and morbidity occurring early after the treatment.^2^, ^3^ The toxicity of the conditioning regimen, the infections, and the immune reactions have a direct impact on the endothelium, which may act as a pathophysiological substrate for aGVHD.^4^, ^5^

Acute GVHD is caused by alloreactivity, defined as the donor‐immunocompetent cell response against foreign recognized molecules of the host's minor and major histocompatibility complex.^6^ Different stages have been proposed for aGVHD.^7^ The first stage involves the effect of the conditioning regimen and the underlying disease, causing the activation of the recipients' antigen‐presenting cells (APC), among which endothelial cells (ECs) can be included, as well as the release of cytokines and chemokines from the injured tissues.^8^ In a second stage, APC promote the proliferation and differentiation of donor T lymphocytes into CD4+ and cytotoxic T‐cell lymphocytes, leading to a third stage in which there is an activation of cellular and inflammatory effectors that cause enhanced injury in the host target tissues and organs, including the endothelium. The pathophysiology of aGVHD implies multiple tissue and organ damage, mainly affecting the liver, the gastrointestinal tract and/or the skin.^9^

Due to their location, ECs exhibit a dual role in aGVHD since they may act as APC and as target organ. In this regard, an increased percentage of apoptotic Casp3+ blood vessels were found in duodenal and colonic mucosa biopsies of patients with severe aGVHD.^10^ Also, in murine experimental aGVHD, severe microstructural endothelial damage and reduced endothelial pericyte coverage with decreased expression of endothelial tight junction proteins were observed in aGVHD target organs. Moreover, in intestinal aGVHD, colonic vasculature structurally changed, with increased vessel branching and vessel diameter.^10^ Altogether indicate that endothelial dysfunction and damage could be involved in the development and severity of aGVHD.

Steroids are the gold standard for the treatment of aGVHD,^11^ due to their potent anti‐lymphocyte and anti‐inflammatory activity. However, a high proportion of patients (20%–25%), especially those at more severe stages, do not respond to treatment with high‐dose systemic steroids, exhibiting very high mortality.^10^, ^12^ No standard treatment for steroid‐refractory aGVHD (SR‐aGVHD) is currently available, and its pathobiology is poorly understood, thereby hindering the development of novel therapeutic approaches.^13^, ^14^ Recent data demonstrated an association between endothelial vulnerability, endothelial damage, and steroid refractoriness.^15^, ^16^, ^17^ In human biopsies and murine tissues from SR‐aGVHD, extensive tissue damage with low levels of alloreactive T cell infiltration in target organs was found. These results provide the rationale for T cell independent SR‐aGVHD treatment strategies,^10^ with special attention to endothelial damage as a target in this setting.

The present pilot study aimed at performing a comparative analysis of the endothelial proteomic profile in response to serum from patients with aGVHD refractory and sensitive to steroids, independently of the prophylactic treatment received, to find differentially expressed proteins that could explain the involvement of the endothelium in the lack of response to steroid treatment. The use of conventional protein identification techniques has intrinsic limitations to advance this research. A combination of nanoliquid chromatography coupled with mass spectrometry and database search strategies could allow a more comprehensive characterization of distinctive EC damage in refractory and sensitive aGVHD.

## 2 MATERIALS AND METHODS

### 2.1 Experimental design

Prospective and observational investigation performed to compare the endothelial proteomic profile in healthy ECs in culture in response to serum samples from steroid‐refractory aGVHD (SR‐aGVHD) and steroid‐sensitive aGVHD (SS‐aGVHD) patients. Blood samples from aGVHD patients, refractory (unfavourable response, *n* = 4) and sensitive (favourable response, *n* = 8) to steroids, were collected at aGVHD diagnosis (before steroid treatment initiation). Patients were grouped by SR‐aGVHD and SS‐aGVHD depending on their response after 15 days on steroid treatment (1–2 mg/kg daily). Endothelial cell (EC) cultures were exposed for 48 h to patients' serum (SR‐aGVHD and SS‐aGVHD treated). ECs were then lysed, and the resulting samples were analysed by nanoliquid chromatography (nanoLC) coupled to mass spectrometry (LTQ‐Orbitrap Velos Pro, ThermoFisher Scientific, Waltham, MA, USA). Protein identification and quantification were performed using isobaric labeling techniques (Proteome Discoverer v.1.4.0.288, ThermoFisher Scientific). Four out of the 10 proteins with higher fold change were validated in cell cultures, by using immunofluorescence (IF) techniques. These proteins were selected based on their superior fold change, independently of their tendency. Their potential functional relation with the endothelium, haemostasis, and cell relevant processes, was also considered.

### 2.2 Patient selection and sample collection

Of the 67 patients who underwent allo‐HCT at the Hospital Clínic de Barcelona (Barcelona, Spain) between June 2019 and September 2020, 12 patients were included in our study. These 12 patients, who underwent allo‐HCT for malignant haematological diseases, were included in the study because they developed aGVHD II‐IV and the collection of pre‐corticotherapy samples (*n* = 12) was possible.

Blood samples from the patients included in the study were collected at aGVHD diagnosis (before initiating steroid treatment) to obtain serum samples by centrifugation (3000 *g* for 15 min). Serum samples were kept at −40°C until use. This study was approved by the Ethics Committee of the Hospital Clínic de Barcelona (HCB/2018/0957) and conducted following standards set forth by the Declaration of Helsinki. The protocol used received institutional review board approval, and all participants provided signed informed consent.

### 2.3 Proteomic analysis

Before proteomic analysis, cell samples were lysed and proteins were extracted by Radioimmunoprecipitation Assay (RIPA) following manufacturer's protocol (ThermoFisher Scientific).

Thirty μg of total protein were reduced with 4 mM 1,4‐Dithiothreitol for 1 h at 37°C and alkylated with 8 mM iodoacetamide for 30 min at 25°C in the dark. Afterwards, samples were overnight digested (pH 8.0, 37°C) with sequencing‐grade Trypsin/Lys‐C Protease Mix (ThermoFisher Scientific) at an enzyme: protein ratio of 1:100. Digestion was quenched by acidification with 1% (v/v) formic acid and peptides were desalted on Oasis HLB SPE column (Waters, Milford, MA, USA), before TMT 10‐plex labeling (ThermoFisher Scientific) following manufacturer instructions. A pool of all samples was used to normalize between samples, labelled with TMT‐126 tag and included in each TMT batch to calculate relative protein ratios. The different TMT 10‐plex batches were desalted on Oasis HLB SPE columns before the nanoLC‐MS analysis.

Labelled and multiplexed peptides were fractionated by High pH Reversed‐Phase Peptide Fractionation Kit (ThermoFisher Scientific) according to manufacturer's protocol. Briefly, each plex was sequentially eluted in eight different ratios of 0.1% triethylamine and acetonitrile. Then, the eluted fractions were evaporated to dryness on a SpeedVac and resuspended with 0.1% formic acid for direct nanoLC‐MS/MS analysis. Fractions of each TMT‐plex were loaded on a trap nano‐column (100 μm I.D.; 2 cm length; 5 μm particle diameter, ThermoFisher Scientific) and separated onto a C‐18 reversed phase (RP) nano‐column (75 μm I.D.; 15 cm length; 3 μm particle diameter) (Nikkyo Technos Co. LTD, Tokyo, Japan) on an EASY‐II nanoLC from ThermoFisher. The chromatographic separation was performed with a 90 min gradient using Milli‐Q water (0.1% formic acid) and acetonitrile (0.1% formic acid) as mobile phases at a flow rate of 300 nL/min. Mass spectrometry analyses were performed on an LTQ‐Orbitrap Velos Pro from ThermoFisher by an enhanced FT‐resolution MS spectrum (*R* = 30,000 FHMW), followed by a data‐dependent FTMS/MS acquisition (*R* = 15,000 FHMW, 40% NCE HCD) from the most intense ten parent ions with a charge state rejection of one and dynamic exclusion of 0.5 min.

Protein identification/quantification was performed on Proteome Discoverer (software v.1.4.0.288; ThermoFisher Scientific) by Multidimensional Protein Identification Technology (MudPIT) combining the 8 raw data files obtained from each fraction of the sample. For protein identification, all MS and MS/MS spectra were analysed using the Mascot search engine (v.2.5). The workflow was set up using two different Mascot nodes combing Homo Sapiens database (74,449 entries) and contaminants database (247 entries), both searches assuming trypsin digestion. Two missed cleavages were allowed and an error of 0.02 Da for FT‐MS/MS fragmentation mass and 10.0 ppm for a FT‐MS parent ion mass were allowed. TMT‐10plex was set as quantification modification and oxidation of methionine and acetylation of N‐termini were set as dynamic modifications, whereas carbamidomethylation of cysteine was set as static modifications. The false discovery rate (FDR) and protein probabilities were calculated by Percolator. For protein quantification, the ratios between each TMT‐label against 126‐TMT label were used and quantification results were normalized based on the protein median.

### 2.4 Endothelial cell culture

A human microvascular endothelial cell line (HMEC‐1) (ATCC, Manassas, VA, USA) was used.^18^, ^19^ Cells were grown with medium MCDB131 (Gibco‐BRL, Madrid, Spain), supplemented with fetal bovine serum (FBS, Biowest, Nuaillé, France), L‐glutamine, and penicillin/streptavidin (Gibco‐BRL), EGF (BD Biosciences, Erembodegem, Belgium) and hydrocortisone (Sigma‐Aldrich, Madrid, Spain). ECs were maintained at 37°C in a CO_2_ atmosphere (5%) and used at passages 10–20. Cell cultures were confirmed to be free of mycoplasma.

### 2.5 Immunofluorescence analysis

Selected proteins were assessed by IF. HMEC‐1 monolayers were incubated with the sera from SR‐aGVHD or SS‐aGVHD patients on 18 × 18 mm^2^ coverslips in 6‐well plates (VWR, Radnor, PA, USA) for 48 h. ECs were fixed (4% paraformaldehyde), permeabilized with 0.1% Tween and then incubated (RT, 1 h) with different antibodies to Apolipoprotein D (ThermoFisher Scientific/Invitrogen), Rapamycin‐insensitive companion of mTOR (Abcam, Cambridge, United Kingdom) and Riboflavin kinase (Santa Cruz Biotechnology, Dallas, TX, USA), respectively, or permeabilized with 0.1% saponin and then incubated with the monoclonal antibody Cytochrome C (Santa Cruz Biotechnology) (RT, 1 h). Then, cells were incubated with secondary antibodies conjugated with Alexa Fluor 555, 594 and 488 (Molecular Probes, Eugene, OR, USA), respectively (RT, 1 h). Nuclei were stained with DAPI (Sigma‐Aldrich). Cells were imaged using a microscope (DM4000 B) equipped with fluorescence filters (Leica, Barcelona, Spain). Images were analysed (ImageJ Fiji, Bethesda, Rockville, MD, USA)^20^ and results were expressed as a percentage of the covered area fluorescence.

### 2.6 Statistics

For proteomic analyses, Mass Profiler Professional Software v.14.5 (Agilent Technologies, Santa Clara, CA, USA) was used. A log base 2 transformation was applied to the data for variance stabilization, data range compression and to make the data more normally distributed. Unpaired *t*‐tests were performed using a *p*‐value cut‐off of <0.05 and multivariate analysis such as Partial Least Squares Discriminant Analysis (PLS‐DA) was performed. To identify differential pathways based on protein–protein interaction networks and enrichment analysis, Search Tool for the Retrieval of Interacting Genes/proteins (STRING) database (https://string‐db.org/) was used. Proteins were clustered using a Markov Cluster Algorithm (MCL) with an inflation parameter of 1.4.

For immunofluorescent analysis, data are reported as Mean ± SEM. For the results obtained from validation experiments, a statistical analysis was carried out with raw data following a normal distribution: comparison between two independent groups was performed using Student's *t*‐test for unpaired samples. All statistical analyses were conducted using SPSS statistical software (version 26; SPSS Inc, Chicago, IL, USA). Results were considered statistically significant when *p < 0.05. The expression of each protein in cells exposed to both conditions, SR‐aGVHD and SS‐aGVHD, was explored in four different experiments in duplicate (*n* = 8 for each protein validation).

## 3 RESULTS

### 3.1 Patients' characteristics

Baseline characteristics of patients are described in Table 1. Acute leukaemia and myelodysplastic syndrome were the most frequent transplantation indications (58%). Most patients (75%) had low to intermediate‐risk diseases, as by the refined disease risk index (DRI). All patients received fludarabine‐based conditioning schemes. Myeloablative conditioning (MAC) regimens were administered in most of patients (*n* = 9). aGVHD prophylaxis consisted of schemes that combined methotrexate (MTX) with tacrolimus (TK) or high‐dose post‐transplantation cyclophosphamide (PTCy) with TK. Mycophenolate mofetil (MMF) was administered in addition to PTCy and TK for haploidentical transplantations. ATG or alemtuzumab were not administered.

**TABLE 1:** Patient, transplantation characteristics and initial degrees of aGVHD according to corticosteroid therapy response. aGVHD patients were classified into two groups: favourable response (steroid‐sensitive, SS‐aGVHD, *n* = 8) and unfavourable response (steroid‐refractory, SR‐aGVHD, *n* = 4). Except for “Median age”, data are expressed as number of cases (*n*) and the % with respect to the total number (%).

	All patients *N* = 12	Favourable response *N* = 8	Unfavourable response *N* = 4
Median age; years (range)	45 (30–69)	45 (30–64)	53 (50–69)
Sex; *n* (male %)	8 (66)	5 (62)	3 (75)
Baseline Diagnosis; *n* (%)			
Acute leukaemia/Myelodysplastic syndrome	7 (58)	5 (62)	2 (50)
Chronic myeloproliferative syndromes	2 (17)	–	2 (50)
Chronic lymphoproliferative syndromes	2 (17)	2 (25)	–
Multiple myeloma	1 (8)	1 (12)	–
Disease risk index; *n* (%)			
Low‐intermediate	9 (75)	5 (62)	4 (100)
High‐very high	3 (25)	3 (37)	–
Donor selection; *n* (%)			
HLA MRD	2 (17)	2 (25)	–
10/10 HLA MUD	7 (58)	3 (37)	4 (100)
7/8 HLA MMUD	1 (8)	1 (12)	–
Haploidentical	2 (17)	2 (25)	–
Conditioning regimen; *n* (%)			
Myeloablative (MAC)	9 (75)	6 (75)	3 (75)
Reduced Intensity (RIC)	3 (25)	2 (25)	1 (25)
GVHD Prophylaxis; *n* (%)			
TK+ MTX	3 (25)	3 (37)	–
PTCy/TK	7 (58)	3 (37)	4 (100)
PTCy/TK/MMF	2 (17)	2 (25)	–
Grade II–IV aGVHD; *n* (%)	12	8	4
Skin involvement	9 (75)	6 (75)	3 (75)
TGI involvement	5 (42)	2 (25)	3 (75)
Liver involvement	1 (8)	–	1 (25)
Grade III–IV aGVHD; *n* (%)	7 (58)	3 (37)	4 (100)
Skin involvement	5 (42)	2 (25)	3 (75)
TGI involvement	4 (33)	1 (12)	3 (75)
Liver involvement	1 (8)	–	1 (25)

Abbreviations: GVHD, graft‐versus‐host disease; HLA, human leukocyte antigen; MMF, mycophenolate mofetil; MMUD, mismatched unrelated donor; MRD, matched related donor; MTX, methotrexate; MUD, matched unrelated donor; PTCy, post‐transplantation cyclophosphamide; TK, tacrolimus.

Acute GVHD was scored using the Glucksberg criteria, considering the degree of organ involvement from II to IV. Initial degree details of aGVHD are described in Table 1. Skin was the most affected organ (grades II–IV: 75%) in both groups, followed by gastrointestinal tract (grades II–IV: 42%); only one patient in the SR‐aGVHD group developed hepatic aGVHD. Thirty‐three percent of patients developed multiple organ involvement. No patient on SS‐aGVHD required further treatment for aGVHD or had new flares. Regarding other complications related to endothelial damage, no patient presented veno‐occlusive disease/sinusoidal obstruction syndrome, thrombotic microangiopathy, capillary leak syndrome, or engraftment syndrome. Data was collected retrospectively and updated in October 2022. Among the 12 patients included in the study, the median follow‐up was 22.6 months (range: 4.4–36.2). A total of 4 patients died during the follow‐up, and the estimated 2‐year overall survival (OS) was 64.2%. The main causes of death for SR‐aGVHD group were septic shock (*n* = 1) and relapsed (*n* = 1). Patients in group SS‐aGVHD died of fulminant adenovirus infection (*n* = 1) and relapsed (*n* = 1).

### 3.2 Differential expression of proteins from cultured ECs exposed to SR‐aGVHD versus SS‐aGVHD serum

A total of 3739 peptides were identified in samples. After filtering, a total of 3576 proteins were estimated and used to check sample integrity and variability. To analyse possible differentially expressed proteins in ECs exposed to SR‐aGVHD vs. SS‐aGVHD sera, both groups were compared using an unpaired t‐test. Forty‐four proteins were differentially expressed in a statistically significant manner.

**FIGURE 1:**
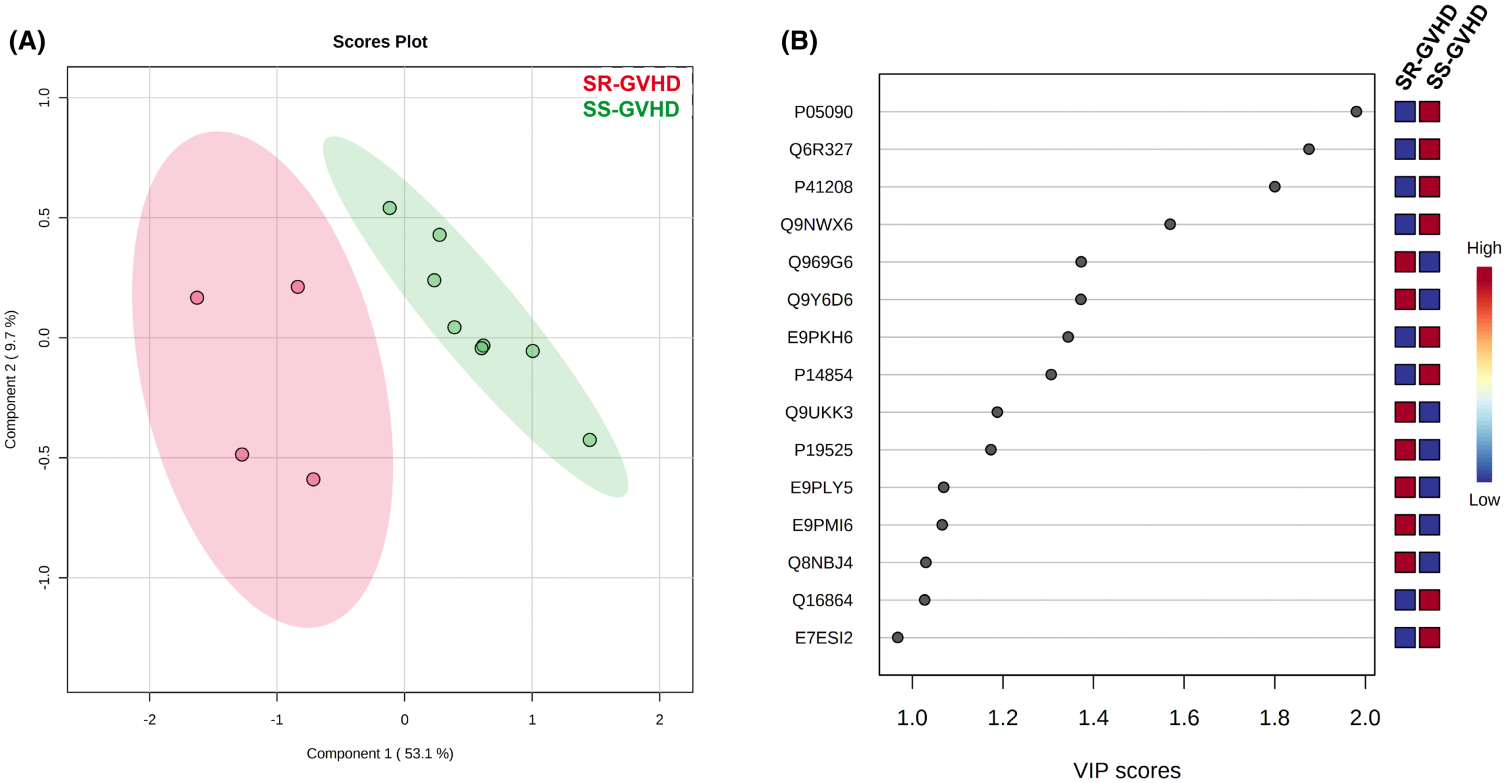
Differential expression of proteins from cultured ECs exposed to SR‐aGVHD versus SS‐aGVHD serum. (A) Partial least squares discriminant analysis (PLS‐DA) scores plot between components 1 and 2. The explained variance is shown in brackets. ECs exposed to sera from steroid‐refractory aGVHD (SR‐aGVHD) patients are presented in red circles and ECs exposed to sera from steroid‐sensitive aGVHD (SS‐aGVHD) patients in green circles. PLS‐DA resulted in a clear separation between the two study groups. (B) Top 15 most important proteins (Swiss‐Prot ID) that contribute more significantly to the separation of two populations in the PLS‐DA model, based on their variable importance in projection (VIP) scores. The right heatmap shows the mean intensity variable in the respective group, with red and blue indicating high and low protein levels, respectively.

The 10 most differentially expressed proteins are shown in Table 2, listed by their fold change (FC > 1.2) independently of their tendency. Considering a significant contribution of these proteins, the scores plot (Figure 1A) showed a clear separation between the two groups of our study (SR‐aGVHD and SS‐aGVHD).

**TABLE 2:** The most differentially expressed proteins when comparing the proteome of endothelial cells exposed to sera from steroid‐refractory aGVHD (SR‐aGVHD) vs. the proteome of cells exposed to sera from steroid‐sensitive aGVHD (SS‐aGVHD) patients. Proteins are listed by their fold change (FC), independently of their tendency (regulation: SR‐aGVHD/SS‐aGVHD). Proteins selected for validation studies appear in grey.

Gene	Swiss‐Prot ID	Protein Name (acronym)	*p*‐value	Regulation	FC
APOD	P05090	Apolipoprotein D (APOD)	0.0383	Down	1.437
RICTOR	Q6R327	Rapamycin‐insensitive companion of mTOR (RICTR)	0.0372	Down	1.417
CETN2	P41208	Centrin‐2 (CETN2)	0.0438	Down	1.350
THG1L	Q9NWX6	Probable tRNA(His) guanylyltransferase (THG1)	0.0445	Down	1.344
RFK	Q969G6	Riboflavin kinase (RIFK)	0.0188	Up	1.289
NDUFS8	E9PKH6	NADH dehydrogenase (ubiquinone) iron–sulfur protein 8 (E9PKH6)	0.0218	Down	1.280
COX6B1	P14854	Cytochrome c oxidase subunit 6B1 (CX6B1)	0.0427	Down	1.255
ARFGEF1	Q9Y6D6	Brefeldin A‐inhibited guanine nucleotide‐exchange protein 1 (BIG1)	0.0112	Up	1.244
PARP4	Q9UKK3	Protein mono‐ADP‐ribosyltransferase PARP4 (PARP4)	0.0047	Up	1.219
EIF2AK2	P19525	Interferon‐induced, double‐stranded RNA‐activated protein kinase (E2AK2)	0.0008	Up	1.200

Several identified proteins appeared downregulated in the ECs exposed to the serum samples from SR‐aGVHD patients (Figure 1B). These proteins were Apolipoprotein D (APOD), Rapamycin‐insensitive companion of mTOR (RICTR), Centrin‐2 (CETN2), Probable tRNA (His) guanylyltransferase (THG1), NADH dehydrogenase (ubiquinone) iron–sulfur protein 8 (E9PKH6), Cytochrome C oxidase subunit 6B1 (CX6B1), V‐type proton ATPase subunit F (VATF), and Cyclin‐dependent kinase 2 (E7ESI2). As for the upregulated proteins in the SR‐aGVHD treated cells, Riboflavin kinase (RIFK), Brefeldin A‐inhibited guanine nucleotide‐exchange protein 1 (BIG1), Protein mono‐ADP‐ribosyltransferase PARP4 (PARP4), Interferon‐induced, double‐stranded RNA‐activated protein kinase (E2AK2), Microtubule‐actin cross‐linking factor 1, isoforms 1/2/3/5 (Fragment) (E9PLY5), and Methylosome subunit pICln (E9PMI6), and Golgi membrane protein 1 (GOLM1) were identified (Figure 1B).

### 3.3 Network among the proteins identified as differentially expressed

**FIGURE 2:**
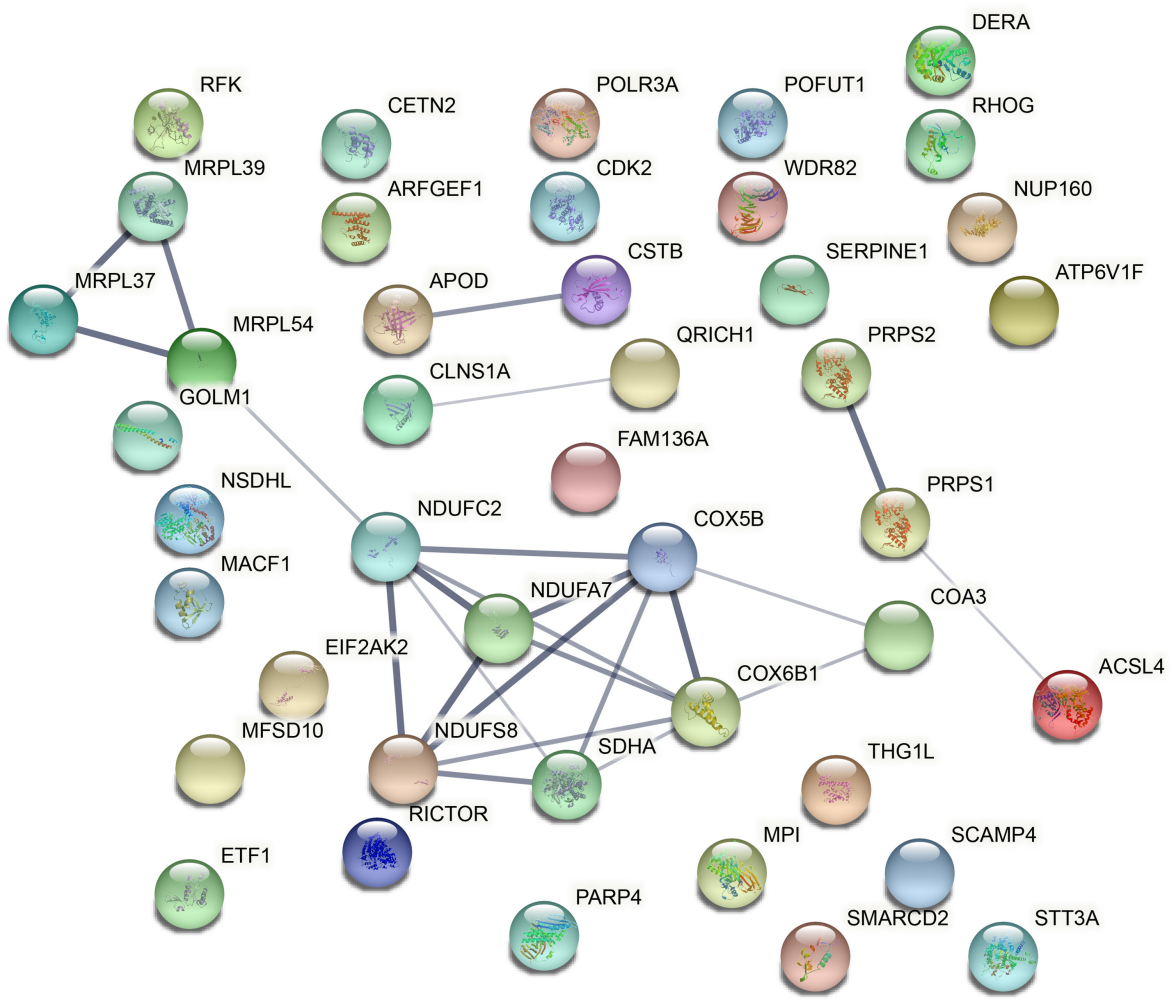
Network among the proteins identified as differentially expressed. Protein–Protein Interaction (PPI) network for these 44 significant proteins obtained in the comparison among ECs exposed to sera from steroid‐refractory aGVHD (SR‐aGVHD) and steroid‐sensitive aGVHD (SS‐aGVHD) patients using STRING database 11.00 (https://strin g‐db.org/). Nodes represent genes that codify its own protein and edges represent interaction between genes (proteins). The thickness of the edge indicates the degree of confidence prediction of the interaction. Only interactions with a high confidence score (>0.7) were considered.

The network generated using the 44 significant proteins identified in the comparison among ECs exposed to sera from SR‐aGVHD and SS‐aGVHD patients is shown in Figure 2. Concerning the most differentially expressed proteins identified, few associations were found. Interestingly, protein E9PKH6 (gene NDUFS8) appears as a hub for many other proteins with similar functions, which are subunits of respiratory Complex I (also known as NADH: ubiquinone oxidoreductase, Type I NADH dehydrogenase and mitochondrial complex I) that is the first large protein complex of the respiratory chains of many organisms, including humans. These are proteins located in the mitochondrial inner membrane. Protein CX6B1 (gene COX6B1) is related to this group of proteins. CX6B1 is also involved in respiratory chains and, as the protein E9PKH6, in oxidative phosphorylation. To note, both proteins are downregulated in SR‐aGVHD.

### 3.4 Validation of the proteomic data by immunofluorescence

The expression of 4 of the most differentially expressed proteins identified after exposure of ECs to sera from SR‐aGVHD and SS‐aGVHD patients was confirmed by using IF in cells exposed to the same conditions as in the proteomic approach. These proteins were APOD, RICTR, RIFK and CX6B1, which were selected based on their significantly higher fold change (independently of their tendency), and their potential functional relation with the endothelium, haemostasis, and cell relevant processes.

**FIGURE 3:**
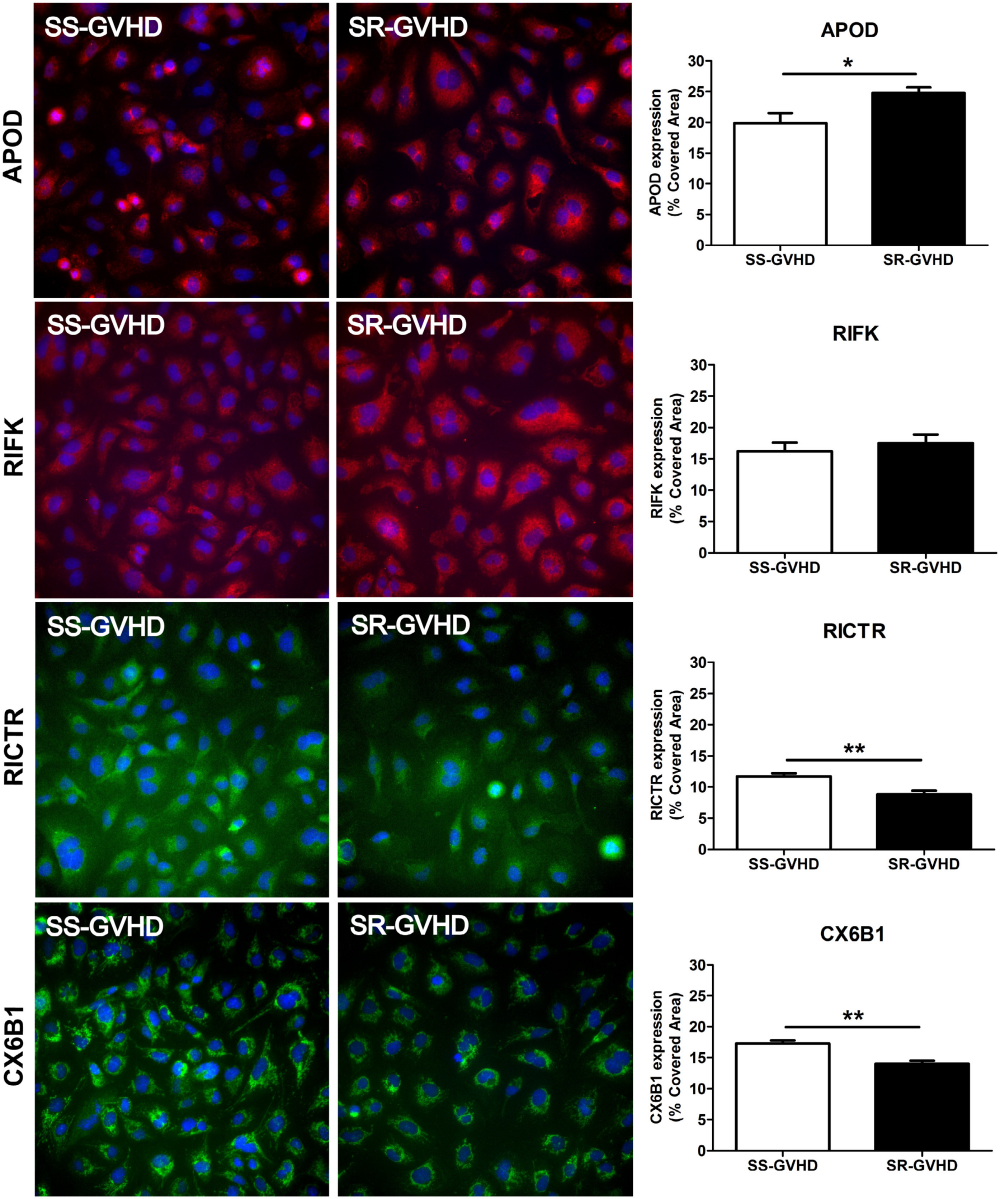
Validation of the proteomic data by immunofluorescence. Representative micrographs of the expression of 4 of the most differentially expressed proteins identified (APOD, RIFK, RICTR, and CX6B1) on ECs in vitro exposed to sera from steroid‐refractory aGVHD (SR‐aGVHD) and steroid‐sensitive aGVHD (SS‐aGVHD) patients. APOD and RIFK are stained in red, RICTR and CX6B1 in green, and the nuclei stained in blue, through immunofluorescence (IF). Bar diagrams represent the quantification of these proteins, respectively. Data are reported in % with respect to the total area of the field analysed, as Mean ± SEM (*n* = 8, being *p < 0.05 and **p < 0.01).

Figure 3 shows the expression on ECs of these 4 proteins (results expressed in % with respect to the total area of the field analysed, Mean ± SEM, *n* = 8). Expression of APOD was significantly higher in ECs in response to SR‐aGVHD serum than in the SS‐aGVHD condition (24.8 ± 0.9% vs. 19.9 ± 1.6%, respectively, *p* < 0.05). The expression of RIFK was increased in response to SR‐aGVHD compared with SS‐aGVHD (17.5 ± 1.4% in SR‐aGVHD vs. 16.2 ± 1.4% in SS‐aGVHD). As for the proteins downregulated in SR‐aGVHD condition, RICTR was found to be significantly reduced in SR‐aGVHD compared with SS‐aGVHD (8.8 ± 0.6% vs. 11.7 ± 0.5%, respectively, *p* < 0.01); and expression of protein CX6B1 was significantly lower in SR‐aGVHD than in SS‐aGVHD (14.0 ± 0.5% vs. 17.3 ± 0.5%, respectively, *p* < 0.01).

Of the 4 proteins validated, APOD was the only one that did not correlate with the proteomic results in terms of tendency. While proteomics indicated a downregulation in SR‐aGVHD ECs, IF showed an increased expression in ECs treated with the same conditions.

## 4 DISCUSSION

**FIGURE 4:**
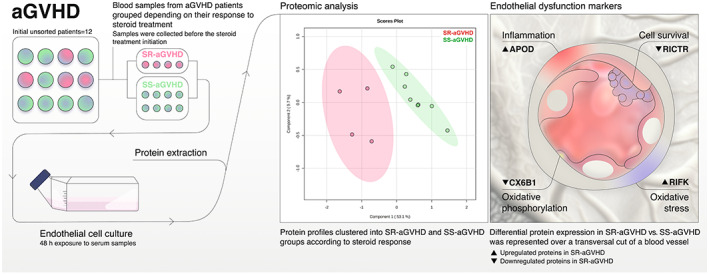
Visual abstract. A concise, pictorial, and visual summary of the main findings of the present study, in which we demonstrate that there is differential protein expression between the endothelial cells exposed to serum from two groups of acute graft‐versus‐host disease (aGVHD) patients depending on their steroid treatment response: steroid‐refractory aGVHD (SR‐aGVHD) and steroid‐sensitive aGVHD (SS‐aGVHD). Differentially expressed proteins validated were: APOD (Apolipoprotein D), RICTR (Rapamycin‐insensitive companion of mTOR), CX6B1 (Cytochrome C oxidase subunit 6B1), and RIFK (Riboflavin kinase).

Despite the curative potential of allo‐HCT, this process is not exempt from complications, among which aGVHD is one of the main, still with high morbidity and mortality. The endothelium is a well‐recognized pathophysiological substrate for aGVHD, acting as APC and target organ. First‐line high‐dose steroid treatment is not always effective. Considering these premises, and by applying a proteomic approach, the main objective of this prospective and observational study was to comparatively analyse the endothelial proteomic profile in response to serum from SR‐aGVHD and SS‐aGVHD patients. Our present results demonstrate that there is differential expression of a variety of proteins. The most prominent changes are related to oxidative phosphorylation and inflammation, downregulated and upregulated, respectively, in ECs exposed to SR‐aGVHD serum samples (Figure 4). Our research provides evidence on proteomic techniques being a reliable and powerful tool to investigate differences in endothelial injury caused by SR‐aGVHD, situation associated with a higher mortality. Some of the newly identified proteins could constitute new biomarkers for steroid resistance in the context of aGVHD.

The incidence of aGVHD has been diminished in association with the administration of high‐dose PTCy.^21^, ^22^, ^23^ The use of PTCy few days after allo‐HCT (days 3 and 4, post‐infusion) is related with better engraftment, low GVHD rates, and non‐relapse mortality in haploidentical HCT. When this strategy is applied in combination with reduced intensity conditioning, it is effective in patients up to 75 years old, with results equivalent to those in younger patients.^24^ For this reason, the use of PTCy combined with other immunosuppressive drugs has been expanded in allo‐HCT in our hospital. As a consequence, the number of patients included in the present study is relatively low, but results could be considered as a proof‐of‐concept.

Due to its location, the endothelium is directly exposed to all the damaging factors occurring during the onset and development of aGVHD. There is endothelial activation and damage in association with allo‐HCT,^25^, ^26^ aggravated with aGVHD. Humoral factors in the sera of patients with aGVHD induce a marked proinflammatory and prothrombotic phenotype in cultured ECs, with increased expression of VCAM‐1 and ICAM‐1 at the cell surface and activation of the intracellular p38MAPK.^27^ Furthermore, there is evidence indicating that angiogenesis plays a major role in aGVHD development,^28^, ^29^, ^30^ with noticeable neovascularization facilitating the migration of inflammatory cells to target organs.^28^, ^31^ In severe aGVHD patients' biopsies, alterations in the endothelium were reported, as well as, in an aGVHD mouse model, endothelial damage was also found.^10^ With respect to SR‐aGVHD, elevated serum levels of different biomarkers of endothelial damage, such as ANG2, were also observed in severe aGVHD patients, not only post allo‐HCT but also pre‐transplantation.^15^

Our present results identify protein APOD, related to inflammation and angiogenesis. In the proteomic analysis, APOD appeared downregulated in ECs in response to SR‐aGVHD samples. APOD is a secreted glycoprotein that has been implicated in governing stress response, lipid metabolism, and aging.^32^ Moreover, APOD is known to regulate smooth muscle cells function and is found in abundance within atherosclerotic lesions. Within blood vessels, APOD is prominently expressed in the perivascular cells surrounding the endothelium during development. It exerts a negative regulation of both cytokine production and T cell migration.^33^ Therefore, APOD seems to act by modulating inflammatory reactions against a variety of stimuli. Also, it appears to be involved in the defence mechanisms against oxidative stress.^34^ Taken together, these observations suggest that APOD could restore the homeostatic balance after injury. Downregulation would imply a lack of protection in front of proinflammatory noxa. However, APOD tendency did not correlate between the proteomic (downregulated) and validation (upregulated) results. We do not have a feasible explanation for this different tendency since cells were treated equally. These results should be studied in a bigger cohort of patients to corroborate its participation. Other proteins related to inflammation, such as PARP4^35^ (also involved in DNA repair^36^, ^37^ and target for several inhibitors developed for cancer treatment) and E2AK2, which regulates NF‐kappaB transcription factor activity,^38^, ^39^ and the oxidative stress‐related protein RIFK^40^ were found to be upregulated in ECs exposed to serum samples from SR‐aGVHD patients. These findings would indicate that the inflammatory and oxidative stress responses may be enhanced in ECs in response to this patients' condition.

One of the selected proteins differentially downregulated in ECs in response to SR‐aGVHD, versus to SS‐aGVHD samples, is RICTR (rapamycin‐insensitive companion of TOR). It belongs to the mTORC2 complex, a group of proteins that contains at least mTOR (target of rapamycin, or sirolimus, which is a macrolide produced by *Streptomyces hygroscopicus* with broad antiproliferative properties)^41^, ^42^, ^43^ and RICTR, in association with other signalling components. mTORC2 complex mediates the phosphorylation and activation of protein kinase B (PKB, also called AKT), implicated in multiple processes such as survival, apoptosis, growth, and proliferation through the phosphorylation of several effectors.^44^ This complex was originally thought to be rapamycin‐insensitive.^45^, ^46^ However, long‐term treatment with rapamycin reduces mTORC2 signalling in some cell types by suppressing mTORC2 assembly.^47^, ^48^ In fact, mTORC2 is involved in cell survival and cytoskeletal organization^45^, ^46^ and RICTR acts as a scaffold protein regulating the assembly and substrate binding of mTORC2.^44^ As RICTR, protein THG1, also related to cell survival,^49^, ^50^ was found to be downregulated in ECs exposed to SR‐aGVHD samples. Therefore, decreased expression of both proteins in ECs exposed to SR‐aGVHD serum samples could indicate that under this condition cell survival is compromised.

Interestingly, two of the most differentially expressed proteins identified, NADH dehydrogenase (ubiquinone) iron–sulfur protein 8 (E9PKH6) and Cytochrome C oxidase subunit 6B1 (CX6B1), are related to oxidative phosphorylation and appeared to be downregulated in ECs exposed to steroid‐refractory serum samples. The network constructed among the proteins identified indicates that E9PKH6 is a hub for many other proteins with similar functions, including CX6B1, which are components of the mitochondrial encoded electron transport chain respiratory Complex I^51^ and Complex IV,^52^ respectively. Oxidative phosphorylation is the primary source of ATP for metabolic and mechanical work, providing most of the energy used for biosynthesis, maintaining proper ion balance, and mechanical work. Therefore, ECs exposed to SR‐aGVHD serum may be metabolically compromised. In addition, downregulation of oxidative phosphorylation has been reported in several malignancies and is associated with poor clinical outcomes.^53^ Furthermore, it is interesting to mention that drug resistance, at least for malignant cells, has been related to mitochondrial function.^54^

BIG1, found to be upregulated in the steroid‐refractory condition, has been related to angiogenesis. It is known that angiogenesis is an early event that arises in aGVHD and develops before immune cell infiltration in target organs.^28^ There is evidence indicating that a depletion of proteins BIG1 and BIG2 inhibits angiogenesis in HUVECs.^55^ According to these results, BIG1 might act by stimulating the formation of new blood vessels by inducing EC proliferation and migration. However, other proteins identified in this study (RICTR) related to proliferation are downregulated in steroid‐refractory conditions, and differences in angiogenesis with respect to the steroid‐sensitive condition should be confirmed. Protein BIG1 is also related to DNA repair and participates in protein synthesis and vesicular transport within the cell.^55^ On the contrary, another protein (CETN2) is also involved in DNA repair^56^ and genome stability,^57^ as well as actin binding and cytoskeleton organization. In our study, CETN2 was expressed downregulated in ECs exposed to steroid‐refractory serum samples.

The present study has limitations, being the small sample size the most important. The difficulty of obtaining a sample just before steroid treatment is initiated, and also the decreasing tendency of GVHD development due to new treatment protocols, including PTCy, are the main causes for the reduced sample size. In addition, the specific role of the underlying malignancies, and the different treatments administered, on the endothelial activation has not been considered here. In fact, the main objective of this study was a proof‐of‐concept demonstration of the involvement of the endothelial damage in response to steroid treatment in aGVHD, and also to prove differential proteomic profiles in steroid refractoriness versus steroid response. Results from the present investigation should be validated in future studies in which different variables could be considered.

Proteomics, as a first‐line experimental technology, results to be a novel approach to differentiate the effect of the SR‐aGVHD and the SS‐aGVHD conditions on ECs. SR‐aGVHD is a complication with high mortality and, thereby, constitutes a major clinical problem. Knowledge of the precise mechanisms that cause the lack of response to steroid treatment is of clinical relevance. Even though the number of samples is limited, the data obtained in this pilot study reinforce that inflammation and angiogenesis are two processes linked to aGVHD, aggravated in response to SR‐aGVHD. The proteomic methodology applied has led us to identify a new cellular pathway, oxidative phosphorylation, downregulated in response to the steroid‐refractory samples, which may act as a differential pathological substrate for this condition. A more exhaustive comparative analysis, including a larger cohort of patients, will allow the validation of the proteins identified as potential biomarkers of response to steroid treatment.

## AUTHOR CONTRIBUTIONS

**Julia Martinez‐Sanchez:** Data curation (equal); formal analysis (equal); investigation (equal); methodology (equal); writing – original draft (equal). **Marta Palomo:** Conceptualization (equal); formal analysis (equal); investigation (equal); methodology (equal); supervision (equal). **Alexandra Pedraza:** Resources (equal); validation (equal); writing – original draft (equal). **Ana Belén Moreno‐Castaño:** Conceptualization (equal); validation (equal); writing – review and editing (equal). **Sergi Torramade‐Moix:** Formal analysis (equal); methodology (equal); software (equal). **Montserrat Rovira:** Resources (equal); writing – review and editing (equal). **María Queralt Salas:** Conceptualization (equal); validation (equal); writing – review and editing (equal). **Joan Cid:** Writing – review and editing (equal). **Gines Escolar:** Conceptualization (equal); supervision (equal); writing – review and editing (equal). **Olaf Penack:** Writing – review and editing (equal). **Enric Carreras:** Conceptualization (equal); funding acquisition (equal); supervision (equal); writing – review and editing (equal). **Maribel Diaz‐Ricart:** Conceptualization (equal); funding acquisition (equal); project administration (equal); supervision (equal); validation (equal); writing – original draft (equal); writing – review and editing (equal).

## FUNDING INFORMATION

Study partially supported by Instituto de Salud Carlos III (FIS PI19/00888), Fundació Marató de TV3 (202026‐10), Deutsche José Carreras Leukämie‐Stiftung (23R/2021), Generalitat de Catalunya (2021‐SGR01118, CERCA), Deutsche Krebshilfe (70113519), Deutsche Forschungsgemeinschaft (PE 1450/7‐1, PE 1450/9‐1) and Stiftung Charité BIH (BIH_PRO_549, Focus Group Vascular Biomedicine).

## CONFLICT OF INTEREST STATEMENT

None of the authors have conflicts of interest directly related to this work. MDR and EC have received honoraria from Jazz. MDR has received research funding from Zacros (Fujimori Kogyo Co., Ltd., Japan), Cellphire Therapeutics (US), CSL Behring (Spain), and Sysmex Europe GmbH. OP has received honoraria or travel support from Gilead, Jazz, MSD, Novartis, Pfizer and Therakos. OP has received research support from Incyte and Priothera. OP is member of advisory boards to Equillium Bio, Jazz, Gilead, Novartis, MSD, Omeros, Priothera, Shionogi and SOBI.

## PATIENT CONSENT STATEMENT

Informed consent to participate in the study was obtained from all individual participants included.

## Data Availability

All authors agree that all data and materials support the published claims and comply with field standards.
